# Fluorescence data of natural organic matter in groundwater from the Fontaine de Vaucluse karst system (2020 to 2021): Excitation-emission matrix (EEM) and 2D spectra at 254 nm excitation wavelength

**DOI:** 10.1016/j.dib.2025.111493

**Published:** 2025-03-21

**Authors:** Leïla Serène, Christelle Batiot-Guilhe, Naomi Mazzilli, Christophe Emblanch

**Affiliations:** a UMR 1114 EMMAH (AU-INRAE), Université d'Avignon, Avignon 84000, France; b HSM, University Montpellier, CNRS, IRD, Montpellier, France

**Keywords:** Humification index, PARAFAC model, Unsaturated zone, Carbonated aquifer, Water quality

## Abstract

This dataset provides bimonthly fluorescence data from February 2020 to October 2021 at 4 springs, 1 loss and 9 unsaturated zone flows of the Fontaine de Vaucluse karst system (France). The data correspond to Excitation Emission Matrices (EEM) and emission spectra at 254 nm excitation wavelength of organic matter fluorescence. Analyses were performed with a SHIMADZU RF-5301 PC spectrofluorometer at the following wavelengths: EEM at λ_ex_= [220; 450] nm, interval=10 nm; λ_em_= [250; 550] nm, interval=1 nm; and 2D emission spectra at λ_ex_=254nm, λ_em_= [250; 530] nm, interval=1 nm. Milli-Q water EEMs are also provided, one for each day of analysis, to allow later correction of the Raman diffraction. Only raw data are provided to allow the user to adapt the corrections to his purpose. The originality of this dataset is the 20-months monitoring of different types of karst waters, from the saturated zone (springs with small or large catchments) to the unsaturated zone which was exceptionally accessible thanks to the Low Noise Underground Laboratory (LSBB, https://lsbb.cnrs.fr/). This dataset was carried out to question the use of the main fluorescence indices (FI, BIX, HIX, [1] and to develop a new index to assess the qualitative groundwater transit time [2,3]. The use of this dataset could be extended to fields other than hydrogeology, such as pedology, microbiology or hydrobiology. It could be usefull to improve the understanding of the carbon cycle as carbon is the main atom constituting organic matter and fluorescence allows the characterization of the molecular structure of the molecule.

Specifications Table

**Table:** 

Subject	Environmental Science
Specific subject area	Fluorescence of natural organic matter in karst groundwater, excitation-emission matrix (EEM) and 2D emission spectra at 254 nm excitation wavelength
Type of data	Raw data corresponding to tables
Data collection	Unfiltered water samples were collected twice monthly from 4 springs, 1 loss and 9 unsaturated zone flows in the Fontaine de Vaucluse karst system between February 2020 and October 2021. Samples collected in dark glass bottles previously precleaned and combusted for 6 h at 550 °C were analysed within a maximum of 3 days using a SHIMADZU RF-5301 PC spectrofluorometer with a 150W xenon lamp, at a stable temperature of 20 °C, slit widths of 15 nm and a fast default scan speed. Instrument stability was checked using the Raman peak of MilliQ water at λ_ex_=348 nm. EEM of Milli-Q water for each day of analysis and from karst waters were acquired at λ_ex_= [220; 450] nm, interval=10 nm; λ_em_= [250; 550] nm, interval=1 nm; and 2D spectra at λ_ex_=254 nm, λ_em_= [250; 530] nm, interval=1 nm.
Data source location	Where the data were collected: • Department: Vaucluse • Region: Provence-Alpes-Côtes d'Azur • Country: France Geographical coordinated of the selected springs and unsaturated zone flows: Entrance of the galleries of the low noise underground laboratory (LSBB, https://lsbb.cnrs.fr/) to access unsaturated zone flows: latitude = 43.928667; Longitude = 5.487102; altitude = 476 m Fontaine de Vaucluse spring: latitude = 43.91954825 ; longitude = 5.13137524 ; altitude = 106 m Millet spring: latitude = 44.15295330; longitude = 5.46306480 ; altitude = 950 m St Trinit spring : latitude = 44.10332400 ; longitude 5.46993500 ; altitude = 830 m Nesque spring: latitude = 44.12518090 ; longitude = 5.41390870 ; altitude = 732 m Nesque loss : latitude = 44.05689130 ; longitude = 5.35569810 ; altitude 625m
Data accessibility	Repository name: OSU OREME [4] Data identification number: (or DOI or persistent identifier) DOI: https://doi.org/10.15148/8d6104e1-ae78-4b4e-8e50-198ccc5b19c9#2024 Direct URL to data: https://data.oreme.org/doi/view/8d6104e1-ae78-4b4e-8e50-198ccc5b19c9#2024 [last access: February, 2025] Instructions for accessing these data: The data can be downloaded directly from the above URL. A table at the bottom of the web page allows clicking on “Fluorescence.zip” to download the data.
Related research article	L. Serène, N. Mazzilli, C. Batiot-Guilhe, C. Emblanch, M. Gillon, M. Babic, J. Dupont, R. Simler, M. Blanc, Questioning calculation and interpretation of fluorescence indices in natural waters studies, Journal of Hydrology. Volume 650, 132524 (2025), ISSN 0022-1694, https://doi.org/10.1016/j.jhydrol.2024.132524.

## 1 Value of the Data

• Few organic matter fluorescence data (EEM and 2D spectra) are presented in data articles, and no datapaper presenting karst groundwater data could be found. Moreover, regular monitoring of natural organic matter fluorescence is unusual and rarely lasts 20 months.
• It is also rare to find both EEM and 2D spectra at λ_ex_=254 nm for the same sample, although the excitation wavelength of 254 nm is mandatory for the calculation of one of the most widely used fluorescence indices, the HIX [5], as the excitation wavelength interval defined for EEMs often omits this wavelength (common interval of 10 or 5 nm, omitting the 254 nm wavelength).
• These data represent a diverse set of karstic waters, including saturated zone waters: springs from small (St Trinit, Millet, Nesque), and large (Fontaine de Vaucluse) catchment areas; as well as unsaturated zone waters. The karst waters of the unsaturated zone are generally monitored from caves, which requires speleological access. Here, access to the underground is granted by an artificial gallery (the low noise underground laboratory LSBB), which allows data to be collected from flows that have no speleological access and are therefore rarely monitored.
• Additional data from these springs and unsaturated zone flows such as discharge, physico-chemical parameters, major ions, total organic carbon are freely accessible [6]; allowing the use of organic matter fluorescence as an additional variable to improve groundwater characterization.
• The data can be useful for several scientific disciplines such as hydrogeology, pedology, microbiology, hydrobiology as organic matter in groundwater mainly comes from the soil, is transported to the aquifer by rainwater infiltration and altered by microorganisms. More broadly, data can contribute to a better understanding of the carbon cycle as carbon is the main atom constituting organic matter and fluorescence allows the characterization of the molecular structure of the molecule.

## 2 Background

Karsts are complex aquifers that are widely used for water supply [7]. Their conduits allow for rapid transit, which is essential to characterize in order to prevent e.g. flooding or overexploitation of water resources. The transit time can be estimated thanks to natural tracers, but few of them are usable in the 0–6-month range. Previous work [8] developed the fluorescence of the natural organic matter as a natural tracer of transit times within this range, using the fluorescence index HIX [9]. This work was based on the unsaturated zone flows of the Fontaine de Vaucluse karst system accessible thanks to the Low Noise Underground Laboratory (LSBB, https://lsbb.cnrs.fr). In order to go further in this research, the same and additional unsaturated zone flows were sampled, as well as springs from the same regional size aquifer. The Transit Time index (TTi) was developed from the spring data and related to the transit time [2] and its applicability to unsaturated zone flows was verified [3]. Finally, a third research paper has been written using all these data to illustrate the questioning of both the calculation method and the interpretation of basic fluorescence indices such as HIX, FI and BIX [1].

## 3 Data Description

### 3.1 Location of the Measuring Stations

**Fig. 1:**
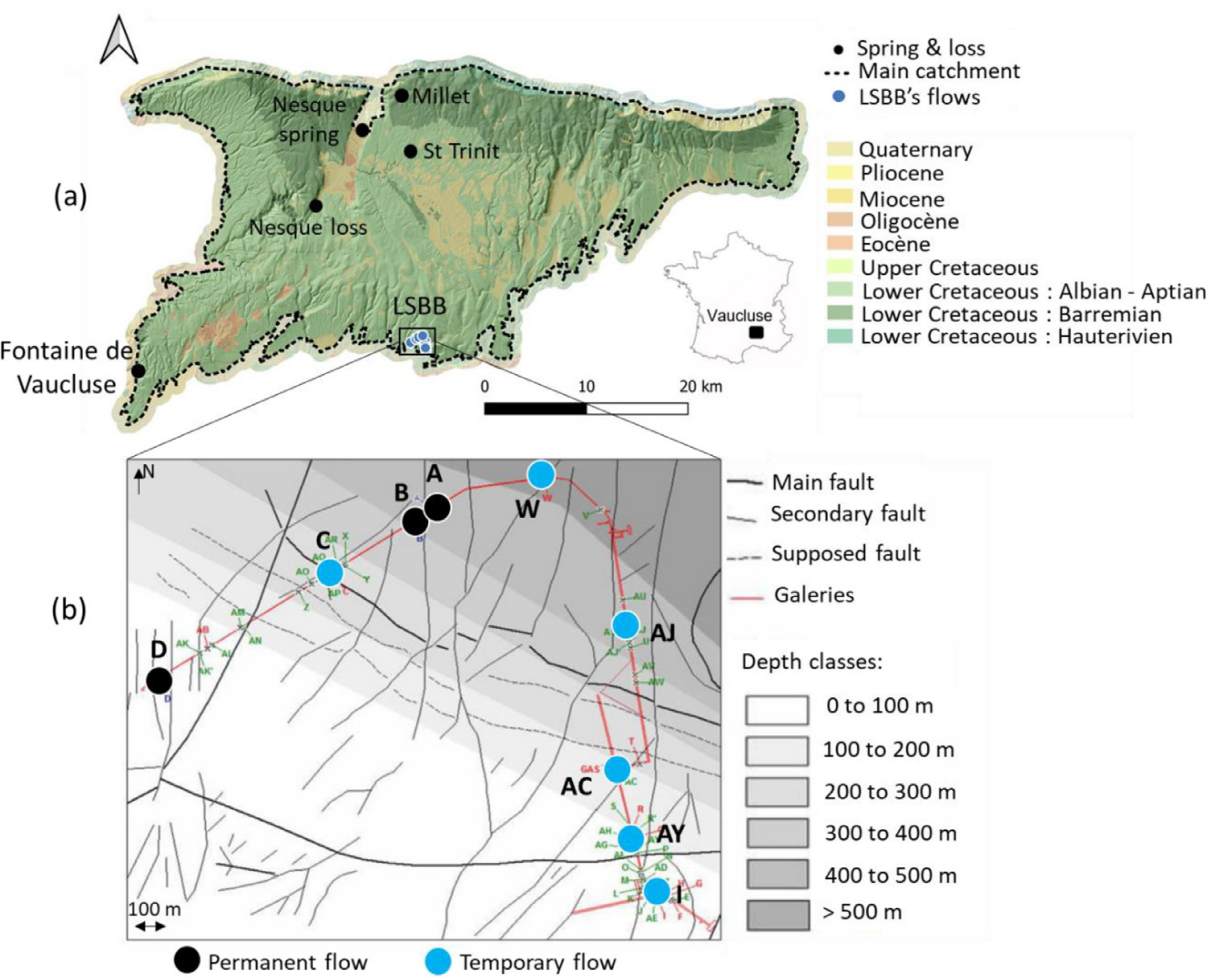
(a) Fontaine de Vaucluse karst system catchment, sampled springs, loss and LSBB location on background of 1:50000 geological map (BD-CHARM from BRGM). Catchment delineation of Fontaine de Vaucluse was taken from [10]*; (b) Position of the unsaturated zone flows sampled on an enlarged view of the LSBB on background of a structural map (modified from* [11]*).*

The study area is located in the Vaucluse karst system in south-eastern France. Fontaine de Vaucluse is the main outlet of the regional size aquifer, and 3 other springs were sampled: St Trinit, Millet and Nesque (Fig. 1a). The Nesque spring is the source of the Nesque river, which flows into a reservoir. The reservoir's overflow disappears into the karst above a weir at the “Nesque loss” site (Fig. 1a). Samples were taken downstream of the weir when the reservoir overflowed.

The unsaturated zone flows sampled were accessed thanks to the artificial galleries of the Low Noise Underground Laboratory (LSBB, https://lsbb.cnrs.fr, Fig. 1). The LSBB galleries are dug in the unsaturated zone of the regional size aquifer. They reach 500 m below the surface. 9 unsaturated zone flows were sampled, some of them are permanent and some temporary (Fig.1b and Table 1).

**Table 1:** Latitude, longitude and depth of the unsaturated zone flows accessed thanks to the LSBB's galleries.

Station	Latitude	Longitude	Depth from surface (m)	Permanent (P) or temporary flow (T)
D	43°56′11.70"N	5°27′57.79"E	33.3	P
C	43°56′22.69"N	5°28′22.67"E	256	T
B	43°56′27.71"N	5°28′34.11"E	421.5	P
A	43°56′28.94"N	5°28′36.93"E	442.2	P
W	43°56′32.00"N	5°28′52.69"E	437.4	T
AJ	43°56′13.97"N	5°29′3.46"E	359	T
AC	43°56′0.62"N	5°29′1.31"E	183.5	T
AY	43°55′51.92"N	5°29′3.10"E	104.1	T
I	43°55′46.85"N	5°29′5.63"E	60.2	T

### 3.2 Available Data

**Fig. 2:**
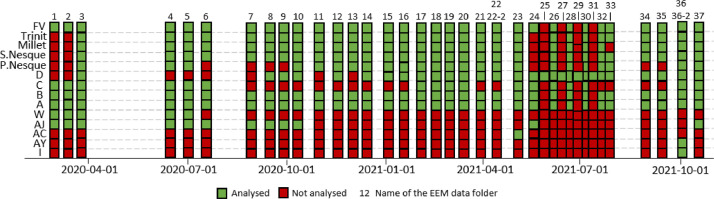
Data availability chart of both EEM and 2D spectra analysis (green boxes). FV correspond to Fontaine de Vaucluse spring, S.Nesque to Nesque spring, P.Nesque to Nesque loss. Green boxes represent each fluorescence of organic matter analysis (EEM and 2D emission spectra at 254 nm excitation wavelength) and red boxes represent the absence of data (no flow, drip only or no sample taken).

The data were collected during 37 sampling campaigns from February 2020 to October 2021 (Fig. 2). The time step is bimonthly, except:

- No sampling during the summer due to laboratory closure, as samples must be analyzed quickly (less than 3 days);
- No sampling from March 2020 to June 2020 because of the sanitary crisis of Covid;
- Weekly time step during the summer of 2021 for the unsaturated zone flow D for a specific research project.

An availability chart is provided in Fig. 2. Missing data may be due to the following reasons:

- Water is not sampled: case of St Trinit, Millet, Nesque springs and Nesque loss for the campaigns 1, 2 and 24;
- Unsaturated zone flow is not flowing or is only dripping: the case of flows D, C, B, A, W, AJ, AC, AY and I when red boxes are present (Fig. 2), except when only flow D was sampled (campaigns 25, 27, 29, 31);
- The water of Nesque reservoir do not overflow: case of Nesque loss at campaign 6, 7, 8, 9;
- The bottle containing the sample was broken or lost: Millet spring, campaign 33.

### 3.3 Location of the Data and Files Names Description

The data can be accessed directly by clicking on the following link, which takes the reader to a web page where the citation, title, abstract, data creator, contributors, editor, metadata, the different versions of the dataset, and conditions of use can be found.

https://data.oreme.org/doi/view/8d6104e1-ae78-4b4e-8e50-198ccc5b19c9#2024

**Fig. 3:**
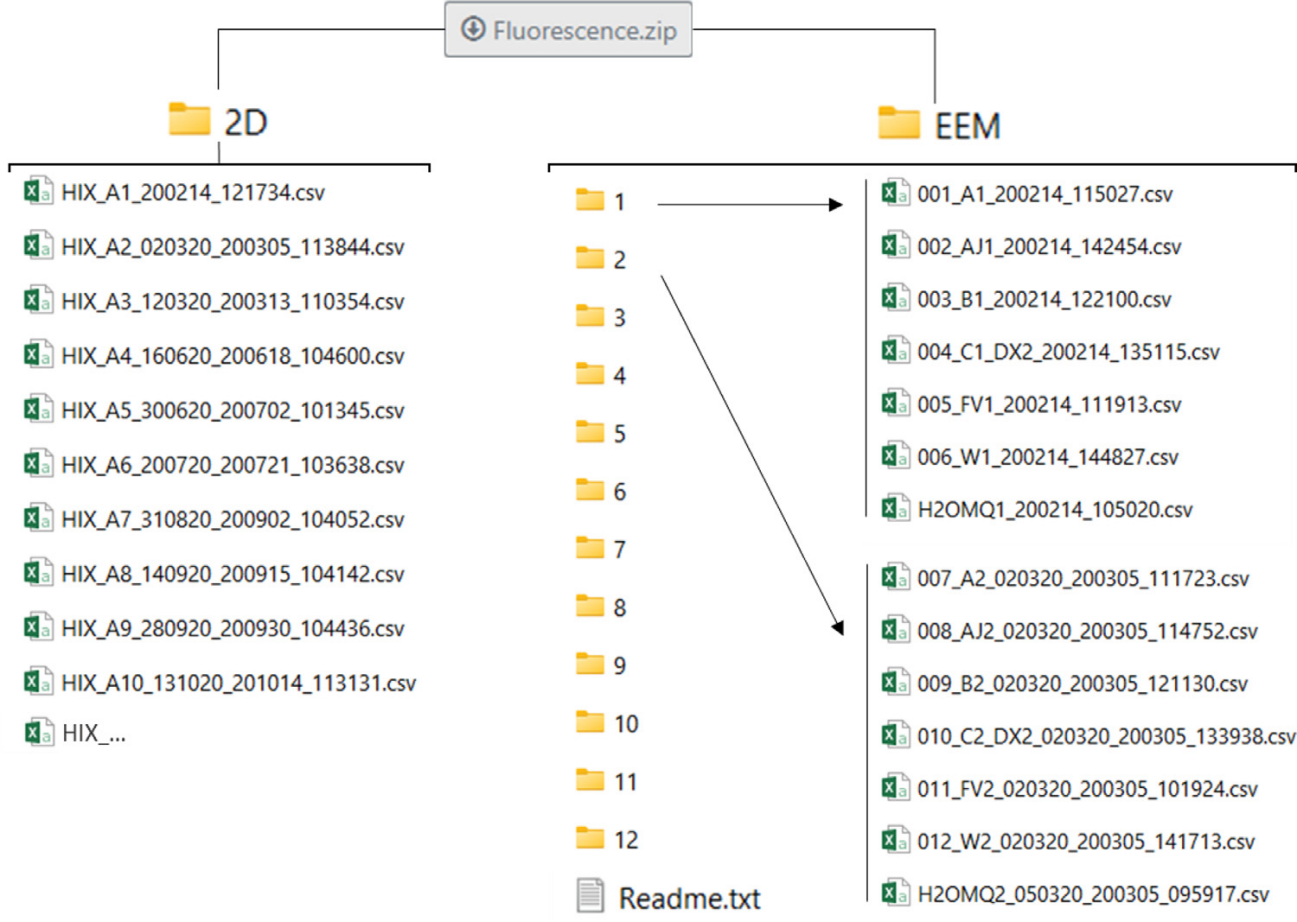
Data organization.

**Fig. 4:**
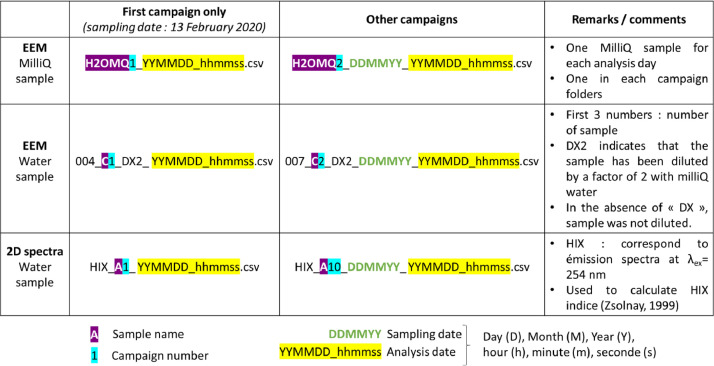
Description of the file's names.

At the bottom of the web page, reader can find a table to download the data in a .zip format. The folder needs to be unzipped to access the data, it is organized as shown in Fig. 3. The 2D folder contains directly the different csv files corresponding to undiluted samples emission spectra at 254 nm excitation wavelength. The EEM folder contains several folders, one for each analysis day. All of them contain one Milli-Q sample (one Milli-Q sample for each analysis day). Sub folders 22, 22-2 and 36, 36-2 correspond to the same field campaign (respectively the 22th and the 36th) but samples were analyzed in two different days. For both 2D and EEM folders, details about the file's names are given in Fig. 4.

## 4 Experimental Design, Materials and Methods

### 4.1 From Bottles Preparation to Sampling

**Fig. 5:**
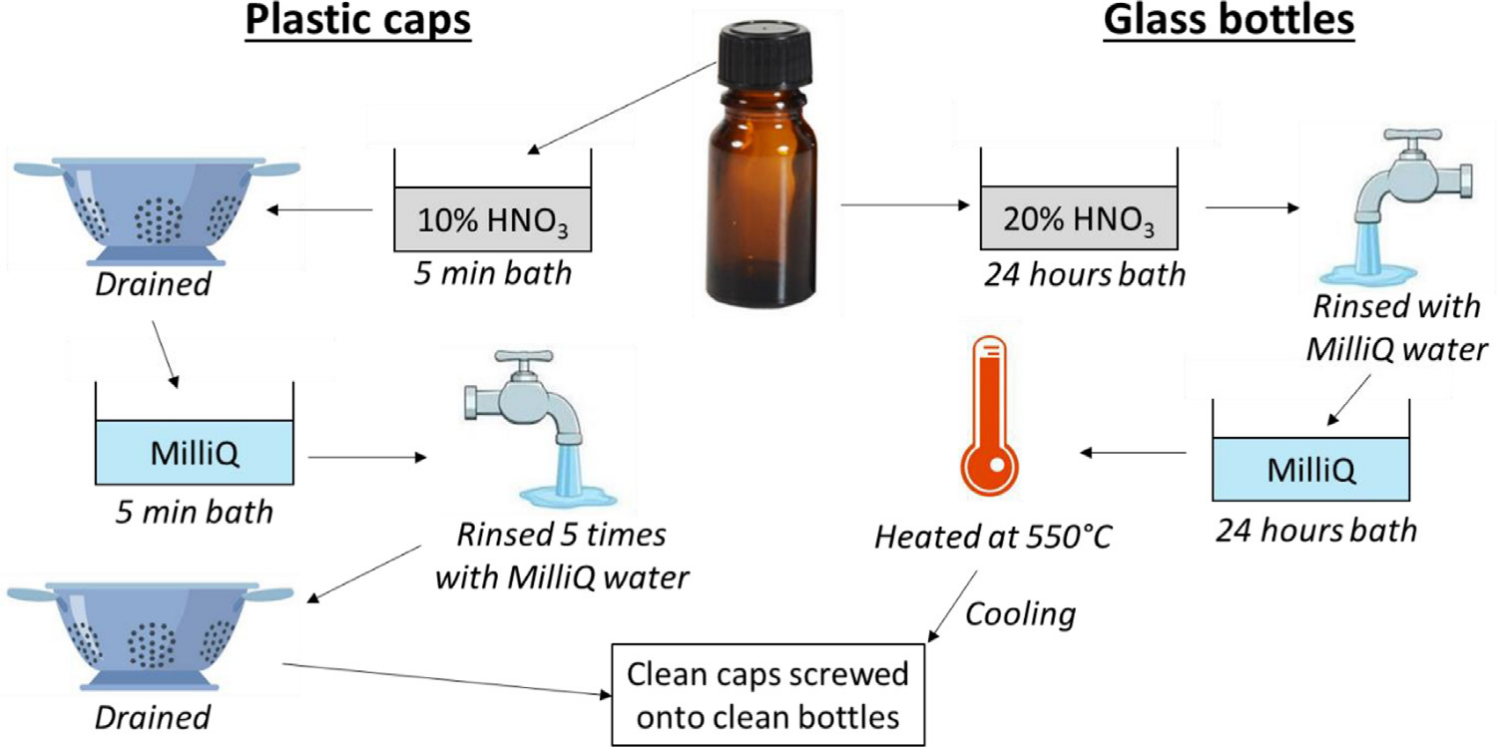
Procedure to clean amber glass bottles and their plastic caps before sampling.

In order not to contaminate the samples, the glass bottles are cleaned of organic matter before sampling (Fig. 5). This is done by immersing the amber glass bottles in a 20% analytical grade nitric acid (HNO_3_) solution for at least 24 h. Each bottle is then rinsed with Milli-Q water and then immersed in Milli-Q water for at least 24 h. Finally, the bottles are heated to 550 °C in a special oven. Once cool, each bottle is sealed with a clean aluminum paper and placed in a plastic bag with a zip.

To clean the plastic caps of the bottles, they are immersed in a solution of 10% analytical grade nitric acid for a maximum of 5 min (Fig. 5). They are then drained in a decontaminated drainer. After draining for a short time, the caps are immersed in Milli-Q water for at least 5 min and then rinsed 5 times before being returned to a clean drainer. To dry them as cleanly as possible, they are covered with clean aluminum paper. Finally, the caps are stored in a clean plastic bag with a zip.

Caps and bottles are reassembled and stored in a clean zipped plastic bag until use. On the field, the glass bottle is rinsed at least 3 times with the sampled water before being filled.

To avoid taking a sample that is not representative of the aquifer, unsaturated zone flow is not sampled if the flow is only dripping.

### 4.2 Samples Analysis

Unfiltered water samples were analyzed no later than 3 days after sampling, thanks to a spectrofluorometer Shimadzu RF-5301PC equipped with a 150 W xenon lamp. Temperature was stabilized thanks to a bath at 20 °C and slit widths of 15 nm were set. Instrument stability was checked using the Raman peak of Milli-Q water at λ_ex_=348 nm, λ_em_=450 nm; and at λ_ex_=350 nm, λ_em_=450 nm. The signal-to-noise ratio was also checked to be above 150 for the Raman of ultrapure water according to the spectrofluorometer manufacturer's instructions.

Before the first analysis and between each sample, the quartz tank was rinsed 3 times with Milli-Q water, then twice with ethanol, and tree more time with Milli-Q water. Extra water outside the tank is swiped with KIMTECH paper.

On each day of analysis, an EEM of Milli-Q water is performed prior to each sample to allow later minimisation of the Raman scattering effect by subtracting the EEMs of pure Milli-Q water from the sample EEMs. These EEMs are performed at the same wavelengths as the samples: λ_ex_= [220; 450] nm, interval=10 nm; λ_em_= [250; 550] nm, interval=1 nm. When a sample reaches maximum intensity, it is diluted and the analysis is repeated until the best dilution is found to allow observation of the different organic matter components.

2D spectra are performed to calculate the HIX fluorescence index according to [9]. Analyses are performed without any dilution at λ_ex_=254 nm, λ_em_= [250; 530] nm, interval=1 nm.

## Limitations

The data presented in this paper are raw data. Blank subtraction, Raman normalization, scatter removal and interpolation are required to correct the data. As these corrections may vary depending on the use of the data, the authors have chosen to provide the raw data to allow users to make their own corrections. For the same reason, samples are not corrected for dilution; for samples affected by this issue, it is mentioned in the file title.

## Ethics Statement

Authors have read and follow the ethical requirements for publication in Data in Brief. This work meets these requirements and authors confirm that the current work does not involve human subjects, animal experiments, or any data collected from social media platforms.

## CRediT authorship contribution statement

**Leïla Serène:** Writing – original draft, Conceptualization, Methodology. **Christelle Batiot-Guilhe:** Writing – review & editing, Supervision, Methodology, Conceptualization. **Naomi Mazzilli:** Writing – review & editing, Supervision, Conceptualization. **Christophe Emblanch:** Conceptualization, Methodology.

## Data Availability

OREMENatural fluorescence of organic matter excitation-emission matrix (EEM) and 2D spectas at 254 nm excitation wavelength of underground water from Fontaine de Vaucluse system (2020 to 2021) (Original data).
